# FAMILY AND SOCIAL NETWORKS FOR AGE-RELATED PLANNING CONVERSATIONS: CHARACTERISTICS AND VARIABILITY

**DOI:** 10.1093/geroni/igz038.2471

**Published:** 2019-11-08

**Authors:** Emily K Chen

**Affiliations:** RAND Corporation, Arlington VA, Virginia, United States

## Abstract

Informal advance care planning (IACP) – that is, conversations with surrogate decision-makers about wishes for health care or end-of-life preferences – have been identified as equally if not more important than legal documentation for achieving a high-quality end-of-life experience. Fairly high rates of adults report having had these conversations; this is especially true of people who are older, sicker, or who have had caregiving experience. However, relatively little is known about the content and characteristics of these conversations, such as who people are talking to, what triggers the conversation, and what is actually said. This paper reports findings from interview-based research that asked 38 middle-age and older adult respondents (ages 55 to 74) about conversations related to several areas of age-related planning, including planning for health care needs and wishes about end-of-life. The interactive interview protocol used the open-source EgoWeb software to elicit information about age-related planning conversations, family and social networks, and who within those networks served as conversation partners for the various topics. We will share results of the analysis of the networks and conversation topics. We found that some individuals more readily engage in discussions about future planning across topics than others, but that part of this is driven by the readiness of their family and friends to engage in these topics. This suggests that targeting individuals to increase rates of IACP has limitations when family and friends are resistant, and that dyadic interventions may be appropriate in some cases.

